# MangoImageBD: An extensive mango image dataset for identification and classification of various mango varieties in Bangladesh

**DOI:** 10.1016/j.dib.2025.111908

**Published:** 2025-07-21

**Authors:** Md Hasanul Ferdaus, Rizvee Hassan Prito, Masud Ahmed, Mohammad Manzurul Islam, Md Sawkat Ali, Muhammad Ibrahim, Ahmed Abdal Shafi Rasel, Maheen Islam, Taskeed Jabid, Md Atiqur Rahman, Md Mizanur Rahoman

**Affiliations:** a Department of Computer Science and Engineering, East West University, Dhaka, Bangladesh; b Department of Agricultural Extension, Ministry of Agriculture, Bogura, Bangladesh; c Department of Computer Science and Engineering, The University of Dhaka, Dhaka, Bangladesh; d Department of Computer Science and Engineering, Begum Rokeya University, Rangpur, Bangladesh

**Keywords:** Machine learning, Computer vision, Image classification, Object detection, Horticulture, Food processing, Precision agriculture, Deep learning

## Abstract

The mango image dataset presented in this article contains clear and detailed images of the fifteen most common and popular mango (*Mangifera indica*) varieties in Bangladesh: Amrapali, Ashshina Classic, Ashshina Zhinuk, Banana Mango, Bari-4, Bari-11, Fazli Classic, Fazli Shurmai, Gourmoti, Harivanga, Himsagor, Katimon, Langra, Rupali, and Shada. The mango specimens were sourced from various fruit markets across six districts of Bangladesh, namely Rajshahi, Chapai Nawabganj, Satkhira, Panchagarh, Rangpur, and Dhaka, which are famous for popular mango cultivation and availability to ensure a wide geographic representation. To maintain the quality and uniformity of images across the dataset, the images were captured using a high-definition smartphone camera under a standardized and controlled environment.

Overall, the full dataset contains a total of 28,515 images, where 5703 images are original (raw) and 5703 images are processed with a blend of both real and virtual backgrounds. The processed images were further augmented resulting in a total of 17,109 augmented images. This is done to enhance their utility for training machine learning and deep learning models, particularly for performing computer vision tasks such as object detection, classification, and segmentation. This augmentation includes transformations such as flipping, rotation, shearing, blurring, variation of brightness and exposure, and introduction of noise to simulate diverse real-world scenarios and improve model robustness.

This dataset holds strong reuse potential across computer vision, agriculture, food processing, and biodiversity research. It supports automated mango variety identification, sorting, grading, and quality assessment in precision agriculture. It can also aid in breeding climate-resilient, high-yield mango varieties, enhancing food security and sustainable farming. Additionally, it facilitates studies on phenotypic diversity, genetic correlations, and regional trait comparisons. The dataset can help ensure traceability, authenticity, and quality assurance, improving supply chains and export potential. From a biodiversity standpoint, it contributes to documenting and conserving unique mango varieties.

Specifications Table

**Table:** 

Subject	Computer Sciences, Agricultural Sciences, Biological Sciences
Specific subject area	Machine Learning and Computer Vision-based automated identification and classification of mango varieties.
Type of data	RGB images (raw and augmented) having 504 × 1120 dimensions in JPEG format.
Data collection	The data collection process started by capturing individual mango photos for each of the mangoes from the fifteen (15) varieties from several angular perspectives utilizing a high-definition smartphone camera. Before capturing photos, each mango is settled on white paper under a white LED light to prevent background-related distortions and color variations in images. The data collection team categorized each mango object and its corresponding image under expert supervision from the agricultural field. Each image was manually checked for quality-related issues, and poor-quality images were filtered out. The MangoImageBD dataset presented in this article has three parts: Original Dataset (*MangoOriginal* folder), Processed Dataset (*MangoRealVirtual* folder*)*, and Augmented Dataset (*MangoAugmented* folder).The data collection processes for each of these parts are discussed below: After the image capture is completed, the non-uniform resolutions of the captured images have been uniformly resized to 504×1120 px by setting several pictures with artificial borders along the width with the help of Python scripts. The brightness of each image is properly adjusted by utilizing Python libraries. Following this process, the Original Dataset is composed of a total of 5703 images. Later, a Processed Dataset is constructed by excluding redundant details from each photo of the raw dataset. The Roboflow online image processing tool has been utilized to annotate the mango with a bounding box to exclude unwanted portions of the images while preserving the whole mango in the resulting image. A total of 250 images have been annotated, which are employed to train an AutoML model on Roboflow to predict the bounding box on every image of the raw dataset. The external parts of the predicted bounding box were replaced with white paddings, and the resulting images were accumulated in the Processed Dataset, resulting in another 5703
	images in this dataset. Finally, various augmentation techniques are implemented and combined on the Processed Dataset images using Roboflow, resulting in a total of another 17,109 images that form the Augmented Dataset.
Data source location	The mango specimens used for capturing images were collected from the following locations in Bangladesh: • Aftabnagar Kacha Bazar, Dhaka, Bangladesh. (23.76540° N, 90.44492° E) • Niketan Bazar, Dhaka, Bangladesh. (23.77313° N, 90.40587° E) • Rangpur City Bazar, Rangpur, Bangladesh (25.75368° N, 89.25344° E) • Lalbagh Bazar, Rangpur, Bangladesh (25.72408° N, 89.25391° E) • Rajshahi Mango Market, Rajshahi, Bangladesh (24.36684° N, 88.59840° E) • Shetu Bazar, Tala, Satkhira, Bangladesh (22.72093° N, 89.07010° E) • Kansat Mango Market, Chapai Nawabganj, Bangladesh (24.74229° N, 88.17248° E) • Mill Gate Bazar, Panchagarh, Bangladesh (26.32731° N, 88.55416° E)
Data accessibility	Repository name: MangoImageBD: An Extensive Image Dataset of Common and Popular Mango Varieties in Bangladesh for Identification and Classification Data identification number: https://doi.org/10.17632/hp2cdckpdr.2 Direct URL to data: https://data.mendeley.com/datasets/hp2cdckpdr/2
Related research article	*None.*

## 1 Value of the Data

• **Research and Computer Vision Development:** The dataset can be utilized for in-depth research on mango variety identification and classification using AI and machine learning. It also serves as a benchmark for testing and improving computer vision algorithms. The diverse visual data supports the development of robust models for detecting subtle variations in features like color, size, and texture.
• **Agricultural Tools and Automation:** This dataset facilitates the development of mobile and IoT-based applications to assist mango farmers and agro-industries in identifying varieties, making harvest decisions, and enhancing productivity. Additionally, it enables automated grading and sorting systems for postharvest handling, ensuring quality control, reducing human error, and minimizing waste across the production line.
• **Supply Chain and Export Optimization:** The dataset supports traceability and transparency by integrating AI-based identification systems into the mango supply chain. This ensures accurate variety verification for meeting international export standards, thereby enhancing the reliability and global reputation of Bangladeshi mangoes.
• **Market and Economic Research:** Businesses can leverage the dataset to study consumer preferences, enabling the design of targeted marketing strategies and more effective distribution of popular varieties. This alignment of production with market demand helps optimize economic outcomes in the mango sector.
• **Biodiversity and Comparative Studies:** By documenting the visual and phenotypic traits of indigenous mango varieties, the dataset contributes to biodiversity research and conservation efforts. It also enables regional and global comparisons, allowing researchers to examine genetic diversity, environmental adaptation, and trait evolution across different geographies.
• **Education and Skill Development:** Serving as a practical educational resource, the dataset supports training for agricultural scientists, horticulturists, and AI researchers. It offers real-world exposure to data-driven methods in agriculture, fostering innovation and capacity building in both academic and industrial settings.

## 2 Background

The motivation for compiling this dataset stems from the growing demand for automated solutions in agriculture and agro-based industries, particularly for fruit identification and classification. Mango (*Mangifera indica*) is a highly valued tropical fruit that plays a significant role in the nutrition, economy, and culture of Bangladesh. The diversity of mango varieties, each with distinct visual characteristics, presents both opportunities and challenges for developing intelligent systems for agricultural management, quality assessment, and variety-specific marketing [1]. However, there are limited publicly available image datasets for mango varieties, especially those native to Bangladesh. Sheikh et al. [2] presented a mango image dataset named Mangifera2012 on Mendeley Data that has only 2012 images of 10 varieties of mangoes in Bangladesh. Compared to the Mangifera2012 dataset, our MangoImageBD dataset has an additional 9 unique varieties of mangoes and comprises a total of 28,515 images, including 5703 original (raw) images and 17,109 sufficiently augmented images to facilitate machine learning (ML) and deep learning (DL) applications. Moreover, the Mangifera2012 dataset has multiple different backgrounds with reflections, whereas our dataset utilizes a single white background without any reflection across all the mango varieties to facilitate uniformity and background noise reduction. Prabhu et al. [3] published a data article for a mere 142 images of Alphonso mangoes in India. Huang [4] uploaded a dataset of 300 original mango images of Chinese mangoes, Ismail [5] uploaded 710 Sala mango images of Malaysia, and Ismail et al. [6] uploaded 294 Harumanis mango images of Malaysia. Islam et al. [7] published a dataset of mango leaves images where they presented the prospect of utilizing AI techniques for detecting mango varieties from images of different mango leaves. Iqbal et al. [8] presented a dataset of eight varieties of mangoes native to Pakistan. Compared to these datasets, our MangoImageBD [9] dataset contains images of different varieties of mangoes that are commonly cultivated and the most popular in Bangladesh.

The data was generated within a framework designed to support computer vision applications in smart agriculture and agro-industries. Images were captured using a high-definition smartphone camera to ensure accessibility and reproducibility. A controlled environment was maintained during image acquisition under standardized conditions such as lighting, background, and positioning, minimizing variability unrelated to the mango samples themselves. Theoretical considerations included the importance of ensuring data diversity to facilitate robust ML/DL model training. To this end, image augmentation techniques were applied by incorporating transformations such as flipping, rotation, shearing, brightness and exposure variation, blurring, and introduction of noise to simulate diverse real-world scenarios and improve model robustness. This methodological approach aligns with the best practices in ML/DL modeling to ensure the dataset meets the requirements for diverse use cases such as classification, object detection, and segmentation.

## 3 Data Description

**Fig. 1:**
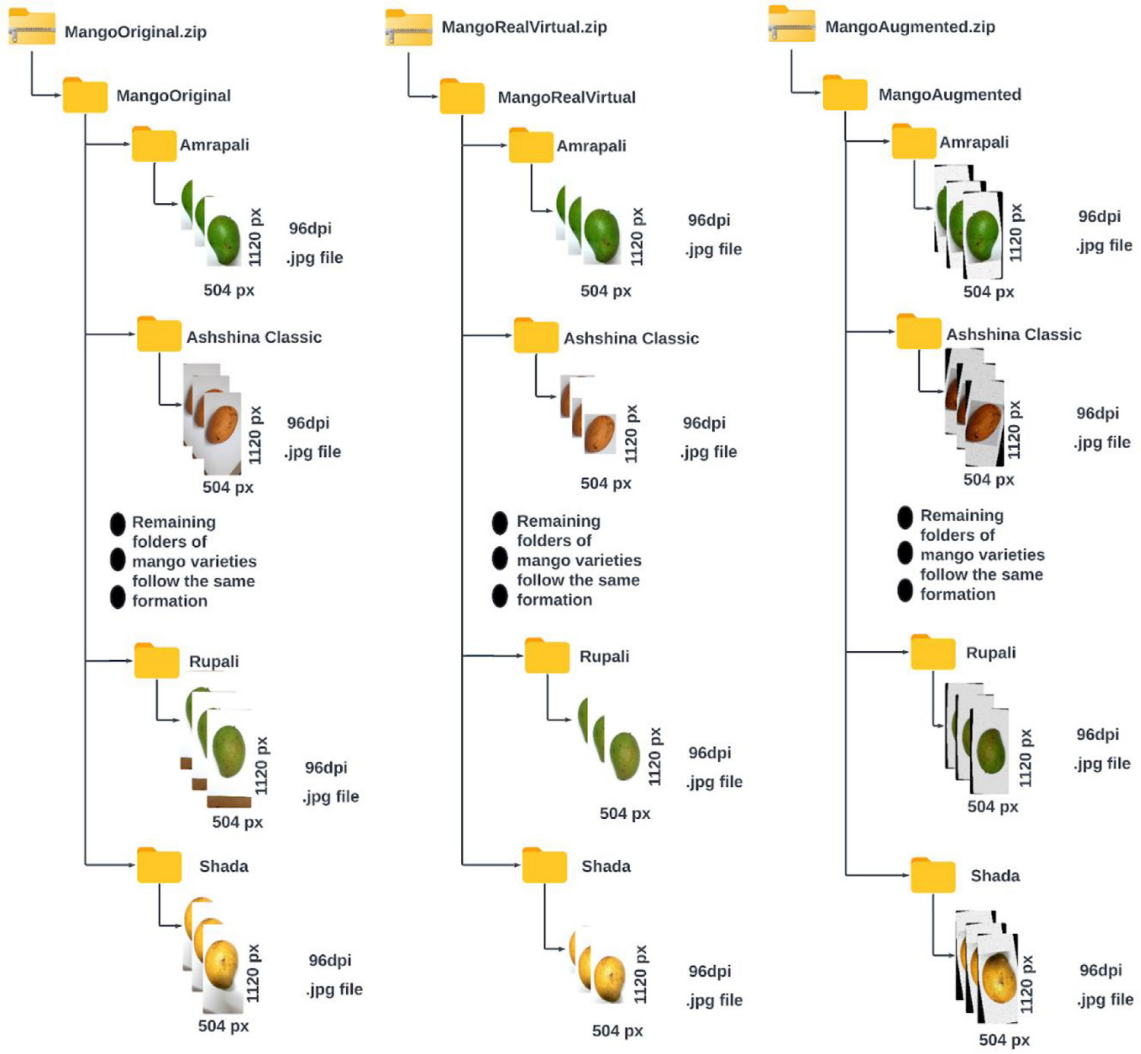
Structure of the main dataset folder.

**Fig. 2:**
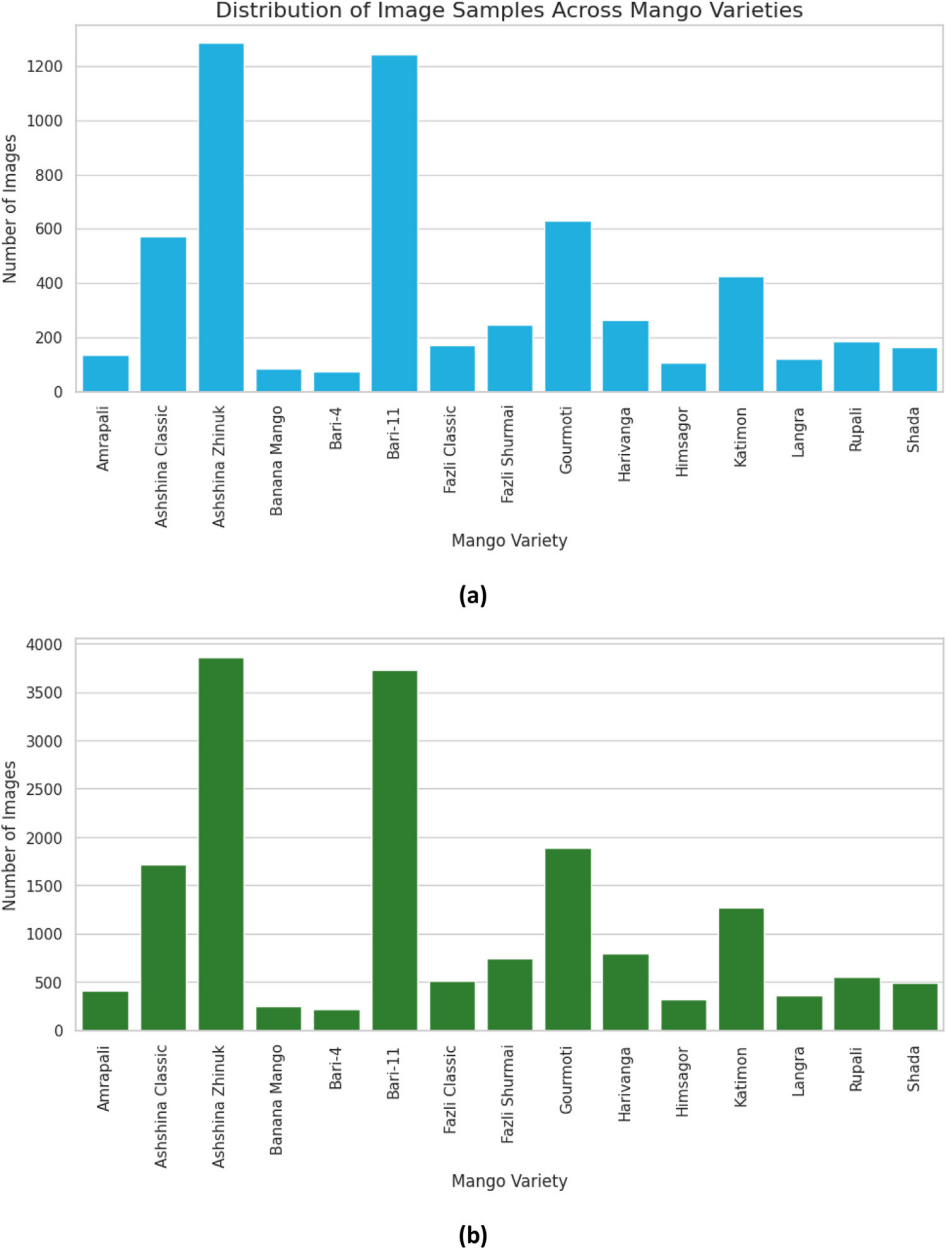
Number of images across mango varieties for (a) Original and Processed Dataset and (b) Augmented Dataset.

Our dataset is organized into three folders - Mango Original folder, *MangoRealVirtual folder*, and *MangoAugmented folder,* representing the three parts of the dataset - Original Dataset, Processed Dataset, and Augmented Dataset, respectively. Each of these folders has fifteen subfolders representing fifteen mango varieties included in our dataset: *Amrapali, Ashshina Classic, Ashshina Zhinuk, Banana Mango, Bari-4, Bari-11, Fazli Classic, Fazli Shurmai, Gourmoti, Harivanga, Himsagor, Katimon, Langra, Rupali, and Shada*. The three datasets comprehensively illustrate the spectacular features of individual mangoes from several angular perspectives and equivalent lighting conditions. Various images of the Original Dataset conveyed controlled white backgrounds, whereas many others portrayed uncontrolled backgrounds. Images of this dataset were captured with varied resolutions. Therefore, to translate the images into a uniform resolution (504×1120 px) while maintaining the aspect ratio of each image, several images are introduced with a white artificial space along the width. Hence, several images of Ashshina Zhinuk and Katimon mangoes, and all of the pictures of Bari-11 mangoes contain white padding above and below them. The Processed Dataset is constructed with the cropped images from the Original Dataset. Cropped images mostly preserved the white portion of the background while keeping the complete shape of the mango. Padding with white spaces is applied around these cropped images to convert them into a uniform resolution of 504×1120 px. The Augmented Dataset bears the resulting images of the implemented image augmentation techniques on the Processed Dataset, including rotation, flipping, shearing, varying brightness, blurring, and adding noise. The whole dataset consists of a total of 28,515 mango images, which are in JPEG format with a resolution of 504×1120 px. Each dataset folder is published on the publicly accessible Mendeley Data repository [9] in compressed zipped form under a Creative Commons Attribution 4.0 International (CC BY 4.0) licence. Under the terms of this license, this dataset can be shared, copied, and modified so long as appropriate credit is given by providing a link to the CC BY license and indicating if any changes are made. The layout of our main dataset folder is illustrated in Fig. 1. Table 1, Table 2 present a summary of the image resolutions, image numbers, and storage sizes of the three parts of the dataset. The distribution of image counts across mango categories for Original, Processed and Augmented Datasets is visualized in Fig. 2. This figure illustrates the presence of class imbalance within the dataset, characterized by a substantially higher number of images for *Ashshina Zhinuk* and *Bari-11*, while classes such as *Banana Mango* and *Bari-4* are significantly underrepresented compared to other mango varieties across all versions of the dataset. Sample images from the three parts of the datasets for each of the fifteen varieties are displayed in Table 3, Table 4.

**Table 1:** Detailed information about the original dataset and processed dataset.

Dataset Name	Mango Variety (Common/ Official Name)	Capturing/ Primary Resolution in pixels (Width x Height)	Converted Resolution in pixels (Width x Height)	Number of Images	Folder Size	Zipped File Size
Original Dataset (Folder Name: MangoOriginal)	Amrapali	1800×4000	504×1120	135	155 MB	135 MB
	Ashshina Classic	1800×4000		571		
	Ashshina Zhinuk	1800×4000, 2992×2992		1286		
	Banana Mango	1800×4000		83		
	Bari-4	1800×4000		74		
	Bari-11	3024×3024		1244		
	Fazli Classic	1800×4000		171		
	Fazli Shurmai	1800×4000		247		
	Gourmoti	1800×4000		630		
	Harivanga	1800×4000		265		
	Himsagor	1800×4000		106		
	Katimon	1800×4000, 2992×2992		424		
	Langra	1800×4000		120		
	Rupali	1800×4000		184		
	Shada	1800×4000		163		
				**Total**	5703	
Processed Dataset (Folder Name: MangoRealVirtual)	Same as the Original Dataset	Varies from image to image	Same as the Original Dataset	Same as the Original Dataset	144 MB	111 MB

**Table 2:** Detailed information about the augmented dataset.

Dataset Name	Mango Variety (Common/ Official Name)	Capturing/Primary Resolution in pixels (Width x Height)	Converted Resolution in pixels (Width x Height)	Number of Images	Folder Size	Zipped File Size
Augmented Dataset (Folder Name: MangoAugmented)	Amrapali	504×1120	504×1120	405	1.06 GB	941 MB
	Ashshina Classic			1713		
	Ashshina Zhinuk			3858		
	Banana Mango			249		
	Bari-4			222		
	Bari-11			3732		
	Fazli Classic			513		
	Fazli Shurmai			741		
	Gourmoti			1890		
	Harivanga			795		
	Himsagor			318		
	Katimon			1272		
	Langra			360		
	Rupali			552		
	Shada			489		
				**Total**	17,109

**Table 3:** Sample images of seven mango varieties from the datasets.

**Table 4:** Sample images of eight mango varieties from the datasets.

### 3.1 File naming conventions

The Original Dataset is organized in the *MangoOriginal* folder. This folder is named to indicate that the images it contains are raw images with no modifications made to the background. The Processed Dataset is kept in the *MangoRealVirtual* folder, where the images have a virtual background generated by removing a part of the original background. Since the images contain both real and virtual backgrounds, the folder is named accordingly. The augmented dataset is stored in the *MangoAugmented* folder, which contains augmented versions of the images, as indicated by its name. Each of these folders has 15 subfolders, named according to the mango varieties. In the subfolders of the *MangoOriginal* folder, the filenames of the images have two parts concatenated with an underscore. The first part is the name of the mango variety, and the second part is a unique number that varies among the images within each subfolder. The subfolders of the *MangoRealVirtual* folder have an additional '_0′ at the end of their image filenames to distinguish them from the filenames of the *MangoOriginal*‘s subfolders. The first two parts of the image filenames of both folders are the same because the images in the *MangoRealVirtual* folder originated from the images in the *MangoOriginal* folder. Images in the subfolders of the *MangoAugmented* folder are generated from the images in the subfolders of the *MangoRealVirtual* folder using the Roboflow web application. As a result, the images in *MangoAugmented*’s subfolders include an additional part in their filenames, assigned by Roboflow, which carries a unique sequence to differentiate each image file. Moreover, two augmented versions are created for each image in the *MangoRealVirtual* folder, and since the original image is also included by Roboflow, a set of three consecutive images (the original and two augmentations) share the same first part of the filename to indicate their relationship. All the folders of each of the three datasets are published as zipped files. Sample image filenames from the *Amrapali* subfolder for each of the datasets are shown below:

MangoOriginal.zip/

│ MangoOriginal/

│ │ Amrapali/

│ │ ├—— Amrapali_1,007,892.jpg

│ │ ├—— Amrapali_1,012,149.jpg

│ │ ├—— Amrapali_1,034,868.jpg

│ │ └—— …

│ └—— …

MangoRealVirtual.zip

│ MangoRealVirtual/

│ │ Amrapali/

│ │ ├—— Amrapali_1,007,892_0.jpg

│ │ ├—— Amrapali_1,012,149_0.jpg

│ │ ├—— Amrapali_1,034,868_0.jpg

│ │ └—— …

│ └—— …

MangoAugmented.zip

│ MangoAugmented/

│ │ Amrapali/

│ │ ├—— Amrapali_1,007,892_0_jpg.rf.1416faead06f152241c2bf248ed507ce.jpg

│ │ ├—— Amrapali_1,007,892_0_jpg.rf.8f8e348ee501114974dafd4e002643f3.jpg

│ │ ├—— Amrapali_1,007,892_0_jpg.rf.c92b3660f013ce0784c2bec7e35c84c8.jpg

│ │ ├—— Amrapali_1,012,149_0_jpg.rf.42cdc66aa6024c05f8a7b5021668b36f.jpg

│ │ ├—— Amrapali_1,012,149_0_jpg.rf.55184b6399dea617c47996ef913b9d4b.jpg

│ │ ├—— Amrapali_1,012,149_0_jpg.rf.6392c290883bf261a65e30c04b0855c5.jpg

│ │ └—— …

│ └—— …

## 4 Experimental Design, Materials and Methods

**Fig. 3:**
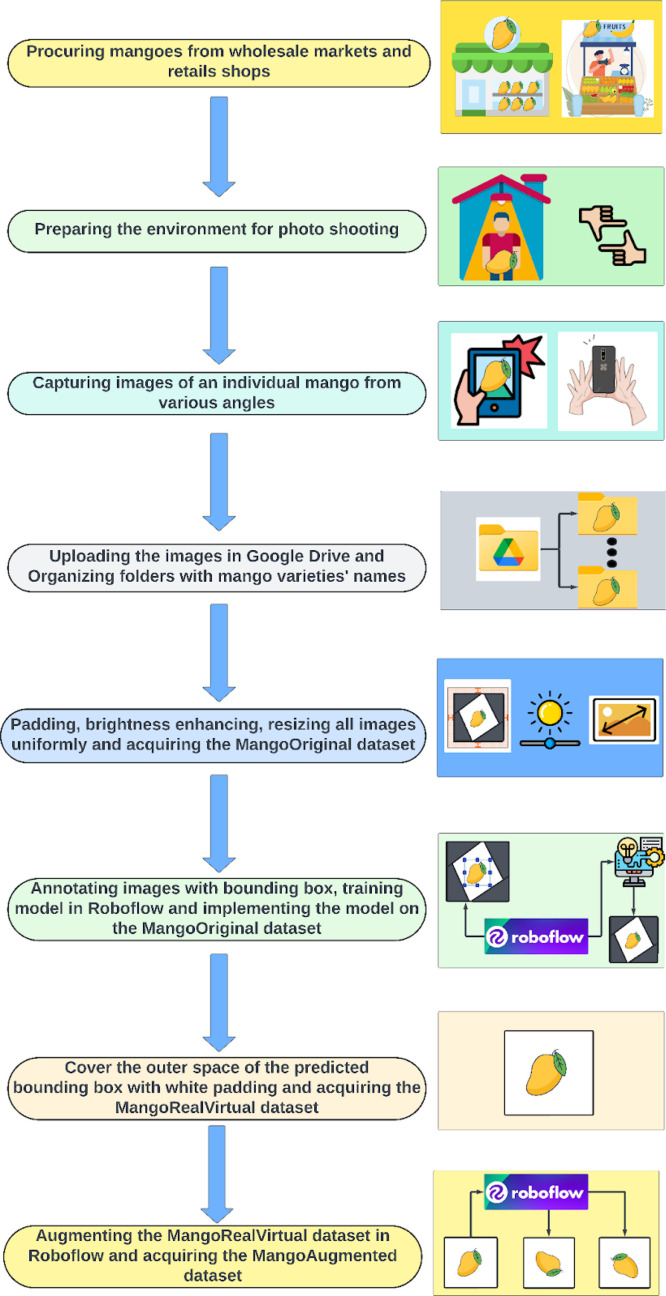
Workflow diagram of the dataset construction process.

The MangoImageBD dataset is constructed using a standard workflow as presented in Fig. 3.

### 4.1 Data source establishment

Mango samples of fifteen diverse varieties were acquired during the mango harvesting season spanning from May to September from the local wholesale and retail fruit markets located in six geographically distributed districts of Bangladesh, namely Chapai Nawabganj, Dhaka, Panchagarh, Rajshahi, Rangpur, and Satkhira, which are renowned for diverse mango cultivation and availability. While obtaining mangoes from markets, only the fresh and unscathed mangoes were prioritized. Images were captured from fresh mangoes before they rot or decay.

### 4.2 Data acquisition

A proper environment was established for capturing the images with maximum details of the mangoes, utilizing a place illuminated by only a white LED bulb while positioning a white paper under each mango specimen, ensuring the actual texture and color of mangoes are illustrated properly and no reflections can appear near the mangoes in the captured images. Images of individual mangoes have been taken from several angular points of view, showcasing the features of shapes and sizes of each type of mango from different views. After photographing one side of the mango, it has been turned around to obtain the color and texture features from the other sides of every mango, making the dataset more reliable for extracting subtle and diverse features from each variety of mangoes. Fostering the diversification in the dataset, every mango was picked only once for photography, indicating that the probability of selecting each mango followed a uniform distribution. Low-quality images were eliminated through real-time visual inspection during the photography session, allowing immediate retakes when necessary to maintain dataset quality. Images have been recorded in several high-resolution dimensions such as 1800×4000, 2992×2992, and 3024×3024 pixels. A detailed specification of the smartphone camera is presented in Table 5. The labels of mangoes are verified by a domain expert agriculturist.

**Table 5:** Specifications of the smartphone camera.

Camera Feature	Feature Specification
Smartphone model	Samsung Galaxy A51
Resolution	48 MP (wide)
Aperture	f/2.0
Focal length	26 mm
Sensor size	1/2.0 inches
Pixel size on the sensor	0.8 µm
Sensitivity to light	ISO 200–400

### 4.3 Data standardization

To organize and manage the images of the fifteen mango varieties, a folder for each type of mango was dedicated. Since ML and DL models are generally trained on images with equal dimensions; hence, images of 2992×2992 and 3024×3024 pixels are horizontally padded with synthetic white portions to convert their aspect ratio to 9:20, as most of the images were in this aspect ratio [10]. Without directly resizing the images with varied dimensions, we have exploited this padding procedure to relinquish image distortion issues while maintaining the same dimension ratio of all images. With the implementation of Python’s image-processing library (*OpenCV*), pictures of all mango categories have been resized to 504×1120 pixels to utilize storage capacity more efficiently while keeping the details in the images intact [10]. Moreover, the brightness of every image is enhanced equally by harnessing the *ImageEnhance* module of Python, enabling the vivid portrayal of features in the images [10]. After executing these procedures, the Original Dataset of 5703 images is obtained, and its compressed ZIP file is uploaded to Mendeley Data [9].

### 4.4 Data annotation

Attaining images of mango samples from various tilted angles introduced several images with unnecessary specifications, inducing any ML/DL model to become ineffective in distinguishing mango varieties. Some effective measures have been followed to deal with this issue. Utilizing the *Roboflow* automation tool, we annotated the mangoes in 250 images by forming a bounding box around the mango while excluding the redundant portions of the picture as much as possible. While generating the bounding boxes, we vigilantly ensured that the boxes did not crop out the shapes of any mango. With the annotated images, we guided an *AutoML* model of *Roboflow* to predict the bounding box on new photos of mangoes. Before training, we partitioned the annotated images into a percentage ratio of 90:5:5 to utilize them as training, validation, and test sets, and augmented the training set to 675 images. Performing the training with an augmented training and validation set, the trained *AutoML* model is evaluated on the test set, where it attained 99.1 % accuracy in predicting bounding boxes. We harnessed the learned model to produce the bounding boxes on the pictures of the Original Dataset. The predicted bounding boxes in each image are explicitly and visually examined to ensure their appropriateness. Few predictions are manually corrected to fit accordingly.

### 4.5 Data refinement

**Fig. 4:**
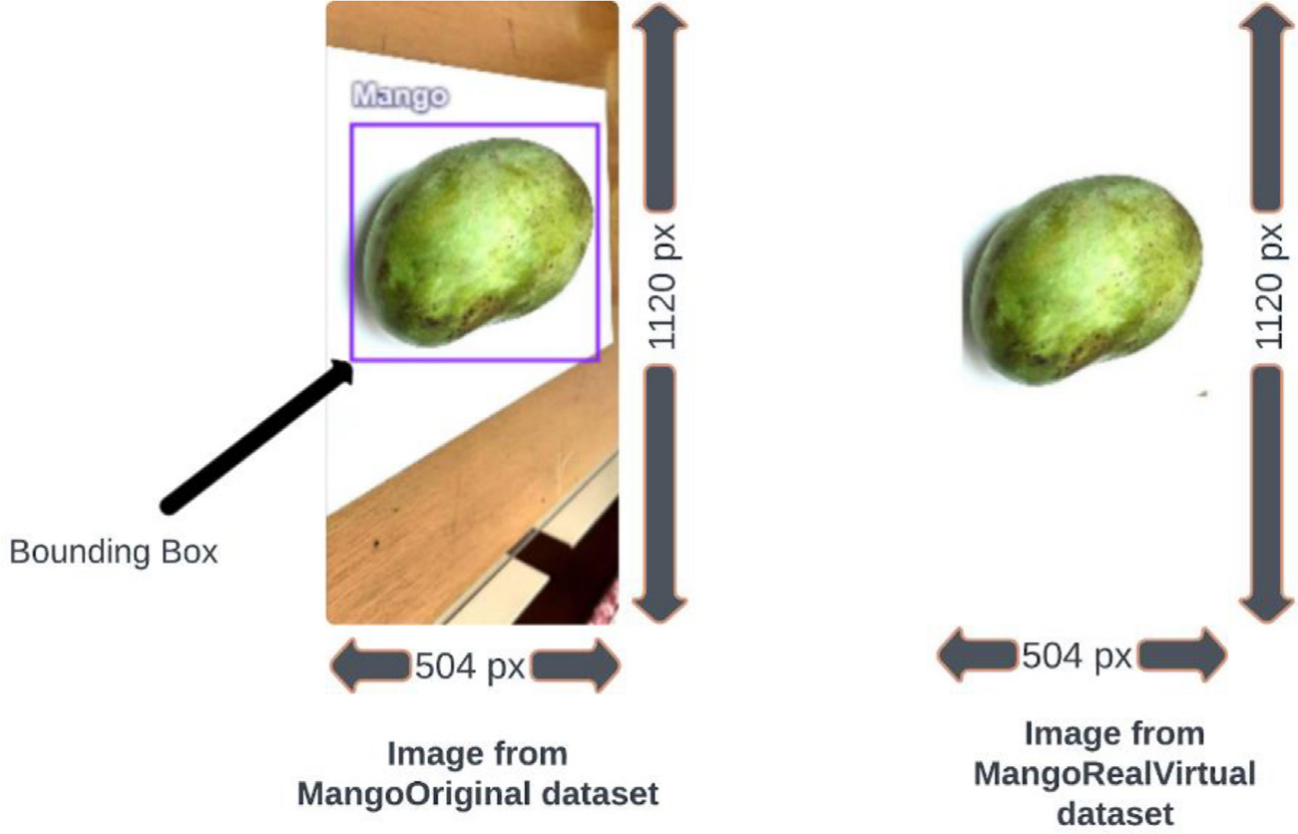
Bounding box annotation in an image (left) in the original dataset and the external space of that bounding box is padded with white areas (right) in the processed dataset.

The external portion beyond the annotated bounding boxes was cropped and filled with white padding using Python scripts using the *Image* module from the Pillow library. Following the padding method, we retained the image dimensions unchanged. Taking advantage of this procedure, unimportant features and background noises in images can be cleaned from any image-based dataset, and that dataset will be more suitable for implementing any ML and DL classification models. At this phase, we acquired the Processed Dataset from our Original Dataset and uploaded it as a compressed ZIP file on Mendeley Data [9]. A sample picture of image annotation with a bounding box and the white padded version of that image is visualized in Fig. 4.

### 4.6 Data augmentation

Augmentation techniques tremendously exhibit their necessity in aiding ML and DL models to effectively improve their learning capabilities in individualizing objects from visual data. These prediction models tend to become overfit on original data during the training period; hence, augmentation procedures extend the amount of data by bringing in controlled distortions to the original data, enabling those models to transform into more resilient and generalized forms to alleviate their overfitting complexity in recognizing new data. Our Augmented Dataset offers a total of 17,109 images for the fifteen mango varieties, which are produced from the 5.703 images of the Processed Dataset. Equipping Roboflow’s automated augmentation option, several augmentation algorithms have been selected following the best practices in the image classification area. While performing the augmentations, multiple techniques are chosen randomly from the determined methods implemented collectively to create one augmented picture. Our proposed Augmented Dataset includes original images from the Processed Dataset, as well as two augmented versions of each original image of the Processed Dataset. The Augmented Dataset is also served as a compressed zipped file on Mendeley Data [9]. Table 6 presents the augmentation methods applied to our dataset and their ranges/types, outcomes, and purposes.

**Table 6:** Specifications of employed augmentation methods.

The selected augmentations were applied to the original images to enhance the diversity and robustness of the image dataset for machine learning and computer vision applications. Horizontal and vertical flipping introduce mirrored versions of mangoes, which help models generalize better to various orientations encountered in practical scenarios. The 90° rotation (upside-down) further ensures robustness against unusual placements or camera angles during image capture. Rotational augmentation within a small range of −10° and 10° simulates slight variations in camera alignment, enabling the model to handle subtle deviations without compromising recognition accuracy.

Shear transformations using angles such as 5°,5°; 5°,−5°; −5°,5°; and −5°,−5° create geometric distortions that mimic perspective shifts often seen when mangoes are photographed from different viewpoints. This helps the model learn invariant features despite such distortions. Brightness adjustments of −15 % and 15 % replicate lighting variations, such as shadows or overexposure, that commonly occur in real-world environments. Similarly, exposure modifications of −5 % and 5 % allow the model to adapt to differences in image intensity caused by varying lighting conditions.

The inclusion of blur augmentation with a 2.5-pixel radius simulates slight camera defocus or motion blur, which prepares the model for real-world image imperfections. Lastly, the addition of noise affecting 2 % of pixels introduces a level of randomness that mimics sensor noise or compression artifacts, further enhancing the model’s resilience to image degradation. Together, these augmentations significantly improve the dataset's ability to train models that perform reliably across diverse and unpredictable visual conditions.

### 4.7 Model evaluation

**Fig. 5:**
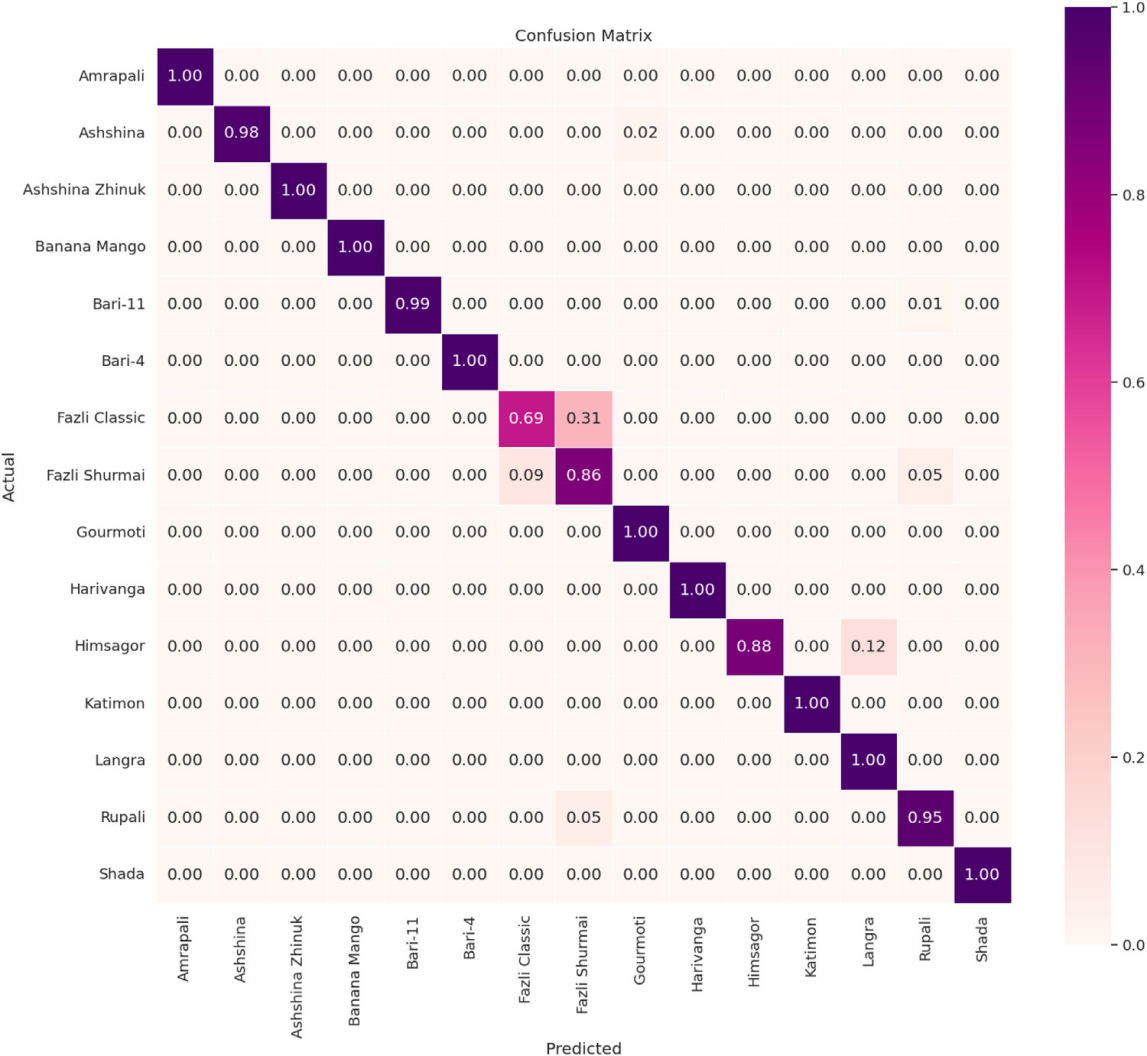
Confusion matrix of the efficientv2b0 model.

A renowned convolutional neural network model, EfficientNetV2B0, is employed on the Processed Dataset to determine the model's mango variety prediction accuracy on our proposed dataset. EfficientNetV2B0 is a lightweight and high-performing CNN architecture designed for speed and efficiency, incorporating fused MBConv blocks and progressive learning techniques to improve training speed, accuracy, and parameter optimization. To apply this CNN model, the dataset is divided into a training-validation-testing section with a ratio of 80:10:10. Images are resized to 224×224 to match the input dimensions expected by the model. The model is trained for 50 epochs with a dropout layer of 40 % added between the global average pooling layer and the dense layer. Imagenet weights have been utilized to harness the pre-training aptitude of this model. Adam optimizer is used with a learning rate of 0.0001. The performance of this model has been evaluated on the unutilized test set in terms of four prominent classification metrics: accuracy, precision, recall, and F1-score. The model achieved 98.07 % accuracy, 95.93 % precision, 95.67 % recall, and 95.73 % F1-score in identifying the 15 categories of mango. In Fig. 5, a normalized confusion matrix is shown to illustrate the model’s ability to recognize each mango category. Table 7 reports the precision, recall, and F1-score obtained for each mango variety. Both Fig. 5 and Table 7 indicate that the model struggled to accurately recognize the Fazli Classic and Fazli Shumari mango classes due to their high similarity in the visual features.

**Table 7:** The precision, recall, and F1-score results of the EfficientNetV2B0 model for each mango variety.

Variety Name	Precision	Recall	F1-score
*Amrapali*	100 %	100 %	100 %
*Ashshina*	100 %	98 %	99 %
*Ashshina Zhinuk*	100 %	100 %	100 %
*Banana Mango*	100 %	100 %	100 %
*Bari-11*	100 %	99 %	100 %
*Bari-4*	100 %	100 %	100 %
*Fazli Classic*	82 %	69 %	75 %
*Fazli Shumari*	79 %	86 %	83 %
*Gourmoti*	98 %	100 %	99 %
*Harivanga*	100 %	100 %	100 %
*Himsagor*	100 %	88 %	93 %
*Katimon*	100 %	100 %	100 %
*Langra*	90 %	100 %	95 %
*Rupali*	90 %	95 %	92 %
*Shada*	100 %	100 %	100 %

## Limitations

• While the dataset was collected in a controlled studio setting using uniform white LED lighting and a white paper background to ensure clarity and consistency, this may slightly limit exposure to natural environmental variations such as diverse lighting or background conditions. Additionally, since the images were captured during a single mango season, there may be a minor seasonal influence on features such as color and texture. However, these are not significant limitations, as the dataset includes a wide range of augmented images that effectively simulate real-world diversity. Augmentations such as flip, rotation, shear, changes in brightness and exposure, blur, and noise introduce valuable variability that help models trained on this dataset perform robustly in diverse and less controlled settings.
• The dataset contains some variation in the number of images per mango variety, ranging from 74 to 1244, resulting in a degree of class imbalance. However, this is a manageable issue in computer vision tasks. Effective strategies such as targeted data augmentation for underrepresented classes and applying weighted loss functions during training can address this imbalance. Additionally, using batch balancing techniques during model training can help ensure fair representation across classes without requiring additional image collection.

## Ethics Statement

The authors have read and followed the ethical requirements for publication in Data in Brief and confirm that the current work does not involve human subjects, animal experiments, or any data collected from social media platforms. All the mango specimens used for image collection were bought from the market, and images were captured under the controlled environment managed by the authors, so there is no requirement for vendor consent, as there are no market photos in the dataset.

## CRediT authorship contribution statement

**Md Hasanul Ferdaus:** Conceptualization, Formal analysis, Data curation, Validation, Writing – review & editing, Supervision. **Rizvee Hassan Prito:** Conceptualization, Investigation, Data curation, Visualization, Writing – original draft. **Masud Ahmed:** Validation. **Mohammad Manzurul Islam:** Data curation. **Md Sawkat Ali:** Project administration. **Muhammad Ibrahim:** Supervision. **Ahmed Abdal Shafi Rasel:** Writing – review & editing. **Maheen Islam:** Writing – review & editing. **Taskeed Jabid:** Supervision. **Md Atiqur Rahman:** Data curation. **Md Mizanur Rahoman:** Project administration.

## Data Availability

Mendeley DataMangoImageBD: An Extensive Image Dataset of Common and Popular Mango Varieties in Bangladesh for Identification and Classification (Original data).

## Appendix Supplementary Materials

Image, application 1
